# ﻿Nycteribiid bat flies (Arthropoda, Insecta, Diptera, Nycteribiidae) of Kenya

**DOI:** 10.3897/zookeys.1169.102800

**Published:** 2023-07-13

**Authors:** Carl W. Dick, Taylor B. Verrett, Paul W. Webala, Bruce D. Patterson

**Affiliations:** 1 Department of Biology, Western Kentucky University, Bowling Green, KY 42101, USA Western Kentucky University Bowling Green United States of America; 2 Negaunee Integrative Research Center, Field Museum of Natural History, Chicago, IL 60605, USA Field Museum of Natural History Chicago United States of America; 3 Department of Biology, University of Oklahoma, Norman, OK 73019, USA University of Oklahoma Norman United States of America; 4 Department of Forestry and Wildlife Management, Maasai Mara University, Narok 20500, Kenya Maasai Mara University Narok Kenya

**Keywords:** Chiroptera, ectoparasites, Kenya, Nycteribiidae

## Abstract

Bat flies (Diptera: Nycteribiidae and Streblidae) are hematophagous ectoparasites of bats characterized by viviparous pupiparity and generally high host specificity. Nycteribiid bat flies are wingless, morphologically constrained, and are most diverse in the Eastern Hemisphere. Africa hosts approximately 22% of global bat biodiversity and nearly one-third of all African bat species occur in Kenya, one of Africa’s most bat-rich countries. However, records of nycteribiid bat fly diversity in Kenya remain sparse and unconsolidated. This paper combines all past species records of nycteribiid bat flies with records from a survey of 4,255 Kenyan bats across 157 localities between 2006 and 2015. A total of seven nycteribiid genera and 17 species are recorded, with seven species from the recent ‘Bats of Kenya’ surveys representing previously undocumented country records. Host associations and geographic distributions based on all available records are also described. This comprehensive species catalog addresses and further emphasizes the need for similar investigations of nycteribiid biodiversity across Africa.

## ﻿Introduction

Bat flies (Diptera: Nycteribiidae and Streblidae) are hematophagous, obligate ectoparasites of bats worldwide. Like other members of superfamily Hippoboscoidea, they are characterized by reproduction via adenotrophic viviparity, wherein a single egg hatches and the larval instars develop within a female, nourished by specialized glands (Dick and Patterson 2006). The primary disassociation of bat flies from their hosts occurs when gravid females use the roost substrate to deposit prepupae; subsequently, flies complete their pupal development off-host and newly eclosed adults must locate and colonize a suitable host.

Bat fly morphology is well-adapted for clinging to the pelage or membranes of bats. Most species of both families possess rows of spiny ctenidia, setae modified into spines, and legs tipped in recurved claws to anchor themselves to their hosts. Many taxa have secondarily evolved winglessness (Theodor 1957a). Bat flies in the family Streblidae possess relatively diverse body plans across the genera, ranging in shape from dorsoventrally flattened to laterally compressed and from wingless to fully flighted. In contrast, members of Nycteribiidae are more morphologically uniform; all species are wingless, dorsoventrally flattened and superficially spider-like, and differ primarily in size rather than shape (Dick and Patterson 2006).

Though both Streblidae and Nycteribiidae are globally ubiquitous, particularly in the tropics and subtropics, nycteribiid bat flies are most diverse in the Eastern hemisphere (Dick and Patterson 2006), with only about 20% of described species occurring in the Western Hemisphere. Nycteribiidae is comprised of 285 species across three subfamilies and 12 genera (Dick and Patterson 2006; Graciolli and Dick 2023). Nycteribiid bat flies may exhibit lower host specificity than streblids (Verrett et al. 2022), but there have been few large and carefully collected surveys of nycteribiids to assess the degree of host specificity across the species. Further, some bat flies have been identified as vectors or hosts of bacterial pathogens and haemosporidian parasites (Megali et al. 2010; Morse et al. 2012; Lutz et al. 2016; Wilkinson et al. 2016), and can harbor viruses related to bat-associated zoonoses (Bennett et al. 2020; Ramírez-Martínez et al. 2021). Therefore, a more complete understanding of nycteribiid diversity and host associations is important for characterizing their role in disease transmission among bats.

Bat biodiversity follows a typical latitudinal trend, with nearly 80% of species concentrated in the tropics (Willig et al. 2003). More than 300 bat species have been described on the African continent (Mammal Diversity Database 2022), of which 104 species have been recorded in Kenya (Musila et al. 2019). Despite Kenya’s position as supporting the richest East African bat fauna (Patterson and Webala 2012), the diversity of nycteribiid bat flies associated with this rich bat fauna remains mostly unexplored. Here we compile all known historical species records of bat flies in Kenya, in addition to identifying and cataloging nycteribiid bat flies from the ‘Bats of Kenya’ survey of 4,255 bats across 157 Kenyan localities between 2006 and 2015. These species accounts contribute to our understanding of the diversity, distribution, and host associations of nycteribiid bat flies in an understudied region.

## ﻿Material and method

**Figure 1.**
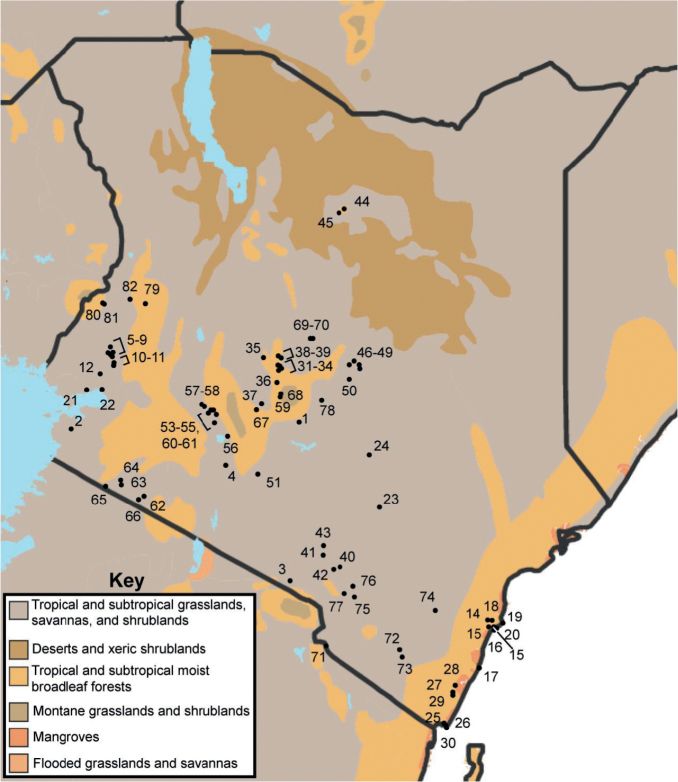
Map of localities sampled in ‘Bats of Kenya’ survey. Gazetteer located in Table 1. Nearby localities with considerable geographic overlap are assigned the same identifying number.

The ‘Bats of Kenya’ surveys were conducted by the Field Museum of Natural History in collaboration with the National Museums of Kenya, Kenya Wildlife Service, Karatina University and Maasai Mara University between 2006 and 2015 (Monadjem et al. 2020) and contributed the bulk of species records in this study. Bats were captured across 157 sampling sites from 82 localities (Table 1), primarily in western, central, and eastern Kenya (Fig. 1). Sampling of foraging and commuting bats was conducted using mist nets erected along trails and roadways, around bodies of water, or at entrance/exit flyways to roosting sites. Additional bats were collected via hand nets within roosts. The properties of roosting sites were variable, ranging from natural (e.g., caves) or relatively permanent anthropogenic structures (e.g., mines, buildings) to more ephemeral roosts (e.g., trees). Following capture, bats were transferred individually to clean cloth bags to minimize parasite disturbance transfers. Bats were euthanized using halothane for collection as museum specimens then fumigated with ethyl ether to ease the extraction of their ectoparasites. Museum specimen collection was performed in accordance with American Society of Mammalogist guidelines (Sikes et al. 2016) and with the approval of the Field Museum’s Institutional Animal Care and Use Committee (most recently 2012-003). Bat flies were immediately transferred to tubes containing 95% ethanol. At the lab, nycteribiid flies were identified using keys and species accounts from Theodor (1967) and via comparison to reference specimens in the Field Museum of Natural History Collection of Hippoboscoid Diptera, where all specimens collected in this survey are currently housed.

**Table 1.** Gazetteer of localities sampled in ‘Bats of Kenya’ survey. Latitude and longitude are presented in decimal degrees. Localities with considerable geographic overlap are assigned the same identifying number.

County	Locality	Locality description	GPS Coordinates
Embu	1	Nyamindi River Cave	-0.556, 37.388
Homa Bay	2	Ruma National Park, Fig. Tree Campsite	-0.646, 34.335
Homa Bay	2	Ruma National Park, houses outside HQ gate	-0.652, 34.343
Kajiado	3	Amboseli National Park, Elephant Research Camp	-2.679, 37.267
Kajiado	3	Amboseli National Park, Amboseli Serena Lodge	-2.705, 37.266
Kajiado	4	Mount Suswa Conservancy, Cave 18A	-1.132, 36.405
Kajiado	4	Mount Suswa Conservancy, Cave 14C	-1.133, 36.402
Kakamega	5	Kakamega Forest, Colobus Circuit 1	0.356, 34.861
Kakamega	5	Kakamega Forest, Colobus Circuit 2	0.356, 34.861
Kakamega	5	Kakamega Forest, Buyangu Kenya Wildlife Service HQ	0.355, 34.866
Kakamega	5	Kakamega Forest, Buzambuli Trail 1	0.350, 34.861
Kakamega	5	Kakamega Forest, Buzambuli Trail 3	0.344, 34.857
Kakamega	5	Kakamega Forest, Ikhondo Campsite	0.352, 34.865
Kakamega	5	Kakamega Forest, Ikhondo Junction	0.353, 34.862
Kakamega	5	Kakamega Forest, Isiukhu River Trail	0.332, 34.879
Kakamega	5	Kakamega Forest, Mukangu Village	0.368, 34.870
Kakamega	5	Kakamega Forest, Kakamega Dam	0.359, 34.866
Kakamega	5	Kakamega Forest, Litali Trail	0.360, 34.861
Kakamega	5	Kakamega Forest, Buyangu Village	0.352, 34.865
Kakamega	5	Kakamega Forest, Buyangu, Glade 1	0.349, 34.870
Kakamega	6	Kakamega Forest, Malava, Edge	0.458, 34.860
Kakamega	6	Kakamega Forest, Malava, Glade 1	0.458, 34.852
Kakamega	6	Kakamega Forest, Malava, Glade 2	0.458, 34.853
Kakamega	6	Kakamega Forest, Kambi ya Mwanza	0.458, 34.853
Kakamega	7	Kakamega Forest, Buyangu Reserve, Edge	0.383, 34.891
Kakamega	7	Kakamega Forest, Buyangu Reserve, Glade 2	0.383, 34.891
Kakamega	7	Kakamega Forest, Kisere Reserve, Edge	0.387, 34.891
Kakamega	7	Kakamega Forest, Kisere Reserve, Glade	0.387, 34.891
Kakamega	7	Kakamega Forest, Kisere Reserve, Kisere Bridge	0.396, 34.883
Kakamega	7	Kakamega Forest, Kisere Reserve, Kisere Farm	0.400, 34.879
Kakamega	7	Kakamega Forest, Mungokho Village	0.375, 34.898
Kakamega	8	Kakamega Forest, Isiukhu River	0.328, 34.880
Kakamega	8	Kakamega Forest, Isiukhu River Trail 1	0.332, 34.879
Kakamega	8	Kakamega Forest, Salazar Trail	0.335, 34.874
Kakamega	9	Mbale	0.096, 34.721
Mbale Vihiga	9	Ilwanda Roost Site	0.097, 34.739
Kakamega	10	Kakamega Forest, Lirhanda Hill Cave	0.218, 34.897
Kakamega	10	Kakamega Forest, Mahiakalo Cave	0.248, 34.906
Kakamega	11	Shamberere	0.378, 34.847
Kakamega	12	Kakamega Forest, Lirhanda Cave	0.212, 34.899
Kilifi	13	Arabuko-Sokoke Forest, Anametra Forest	-3.305, 39.937
Kilifi	13	Arabuko-Sokoke Forest, Brachystegia Forest	-3.282, 39.971
Kilifi	14	Arabuko-Sokoke Forest, Jilore Staff Quarters	-3.216, 39.925
Kilifi	15	Arabuko-Sokoke Forest, Kenya Wildlife Service HQ	-3.300, 39.995
Kilifi	15	Arabuko-Sokoke Forest, 2.1 km NW camp	-3.287, 39.982
Kilifi	15	Arabuko-Sokoke Forest, Pipit Campsite	-3.300, 39.995
Kilifi	15	Arabuko-Sokoke Forest, Gedi Ruins	-3.309, 40.018
Kilifi	15	Arabuko-Sokoke Forest, mango orchard 300 m north of KWS HQ	-3.303, 39.999
Kilifi	16	Gede, Kaboga Cave	-3.335, 40.031
Kilifi	16	Gede, Watamu Cave	-3.351, 40.014
Kilifi	17	Hodihodi Cave	-3.850, 39.810
Kilifi	18	Kakuyuni Dispensary	-3.27, 39.983
Kilifi	19	Malindi Marine Park, Kenya Wildlife Service HQ	-3.255, 40.132
Kilifi	20	Watamu, Makuruhu Cave	-3.323, 40.042
Kirinyaga	21	Sagana Tunnel	-0.117, 34.541
Kisumu	21	Kit Mikayi	-0.117, 34.541
Kisumu	22	Kisumu Impala Sanctuary, Impala Public Campsite	-0.109, 34.746
Kisumu	22	Kisumu Impala Sanctuary, Ecolodge	-0.115, 34.744
Kisumu	22	Kisumu Impala Sanctuary, State Lodge Campsite	-0.110, 34.746
Kitui	23	Mutha, Ngelani Baobab	-1.698, 38.467
Kitui	24	Mwingi, Baobab tree	-0.992, 38.330
Kitui	24	Mwingi, Mutyangome Dam	-0.959, 38.337
Kitui	24	Mwingi, Mwalo Dam	-1.019, 38.326
Kitui	24	Mwingi, Khaluku Rock Dam	-0.992, 38.330
Kwale	25	Fikirini, Pare Cave	-4.590, 39.331
Kwale	26	Fikirini, Three Sisters, Kisimani Cave	-4.615, 39.353
Kwale	26	Fikirini, Three Sisters, Mdenyenye Cave	-4.614, 39.354
Kwale	26	Fikirini, Three Sisters, Pangani Cave	-4.614, 39.354
Kwale	26	Kisite-Mpunguti Marine Park and Reserve	-4.647, 39.378
Kwale	26	Shimoni, “Slave Cave”	-4.647, 39.380
Kwale	27	Kenya Forest Service, Kwale Office	-4.174, 39.452
Kwale	28	Mwaluganje Community Elephant Sanctuary, Ngomeni Cave	-4.082, 39.483
Kwale	29	Shimba Hills National Reserve, Sable Bandas	-4.215, 39.451
Kwale	30	Wasini Island, Wasini Village, Wasini Lodge & Restaurant	-4.658, 39.368
Laikipia	31	Lolldaiga Hills Ranch, Samaki Dam	0.144, 37.115
Laikipia	32	Lolldaiga Hills Ranch, Kambi Dam	0.201, 37.130
Laikipia	33	Lolldaiga Hills Ranch, Konambaya	0.183, 37.146
Laikipia	33	Lolldaiga Hills Ranch, Main gate	0.170, 37.163
Laikipia	33	Lolldaiga Hills Ranch, Main house	0.212, 37.122
Laikipia	33	Lolldaiga Hills Ranch, Farm house	0.201, 37.130
Laikipia	33	Lolldaiga Hills Ranch, Mugumo Campsite	0.170, 37.163
Laikipia	33	Lolldaiga Hills Ranch, Valley Dam	0.172, 37.148
Laikipia	34	Lolldaiga Hills Ranch, Munanda Dam	0.227, 37.117
Laikipia	34	Lolldaiga Hills Ranch, Shaita Dam	0.230, 37.110
Laikipia	34	Lolldaiga Hills Ranch, Shaita Valley	0.228, 37.113
Laikipia	34	Lolldaiga Hills Ranch, Simba Campsite Dam	0.204, 37.105
Laikipia	34	Lolldaiga Hills Ranch, West Kiburuti Borehole Dam	0.188, 37.082
Laikipia	35	Ol Jogi Willdife Conservancy, Kiboko Campsite	0.317, 36.911
Laikipia	35	Ol Jogi Wildlife Conservancy, Ol Jogi Dam	0.325, 36.935
Laikipia	35	Ol Jogi Wildlife Conesrvancy, Pyramid Camp	0.309, 36.076
Laikipia	35	Ol Jogi Wildlife Conservancy, Water Treatment Site	0.304, 36.925
Laikipia	36	Nanyuki, Kenya Wildlife Service Station	-0.015, 37.091
Laikipia	37	Monkey Hills, Mpala Research Centre	-0.308, 36.887
Laikipia	38	Lolldaiga Hills Ranch, Kiburuti Bridge	0.309, 37.150
Laikipia	38	Lolldaiga Hills Ranch, Kiburuti Camp	0.308, 37.152
Laikipia	39	Lolldaiga Hills Ranch, Ngainito Hill	0.338, 37.111
Makueni	40	Chyulu Hills National Park, campground	-2.499, 37.940
Makueni	41	Chyulu Hills National Park, Guano Cave 1	-2.321, 37.693
Makueni	41	Chyulu Hills National Park, Guano Cave 3	-2.340, 37.709
Makueni	42	Chyulu Hills National Park, Kisula Cave	-2.529, 37.853
Makueni	43	Kiboko, Kenya Wildlife Service Station	-2.203, 37.714
Makueni	43	Kiboko, Hunter’s Lodge	-2.211, 37.714
Marsabit	44	Marsabit National Park and Reserve, 1.3km SE campground and headquarters	2.309, 38.000
Marsabit	44	Marsabit National Park and Reserve, campground near headquarters	2.320, 37.994
Marsabit	44	Marsabit National Park and Reserve, Marsabit Lodge	2.309, 37.966
Marsabit	45	Marsabit National Park and Reserve, 12.09km SW of campground	2.235, 37.927
Marsabit	45	Marsabit National Park and Reserve, 6.07km SW of campground	2.283, 37.954
Meru	46	Meru National Park, Bwatherongi Campground	0.165, 38.208
Meru	46	Meru National Park, Kinna	0.170, 38.194
Meru	47	Meru National Park, Kanjoo Gate Compound	0.220, 38.065
Meru	48	Meru National Park, Leopard Rock Lodge	0.223, 38.201
Meru	49	Meru National Park, Murera Gate bandas	0.268, 38.129
Meru	49	Meru National Park, Murera Gate guardhouse	0.268, 38.121
Meru	50	Meru National Park, Ura Gate	0.024, 38.066
Nairobi	51	Karura Forest Cave	-1.250, 36.837
Nakuru	52	Gilgil, Diatomite Cave	-0.430, 36.174
Nakuru	53	Gilgil, Jaika Cave	-0.564, 36.254
Nakuru	53	Gilgil, Kwapotea Cave	-0.564, 36.254
Nakuru	54	Gilgil, Kariandusi Mines	-0.451, 36.282
Nakuru	55	Gilgil, Pipeline Cave	-0.539, 36.294
Nakuru	56	Kenya Wildlife Service, Hippo Camp	-0.742, 36.432
Nakuru	57	Lake Nakuru National Park, Rift Regional Headquarters	-0.306, 36.082
Nakuru	57	Lake Nakuru National Park, Backpackers’ Campsite	-0.317, 36.084
Nakuru	57	Lake Nakuru National Park, headquarters	-0.306, 36.082
Nakuru	58	Lake Nakuru National Park, Lion Hill Cave	-0.346, 36.119
Nakuru	59	Menengai Crater, Mau Mau Cave	-0.217, 37.137
Nakuru	60	Soysambu Conservancy, Field Study Office	-0.392, 36.242
Nakuru	60	Soysambu Conservancy, Serena Elementaita Lodge	-0.403, 36.239
Nakuru	61	Soysambu Conservancy, Monkey Bridge Campsite	-0.392, 36.211
Narok	62	Masai Mara National Reserve, Research Station	-1.547, 35.306
Narok	62	Masai Mara National Reserve, Mara Simba Lodge	-1.547, 35.306
Narok	62	Masa Mara National Reserve, Sarova Mara Lodge	-1.531, 35.320
Narok	63	Mara Conservancy, Eluai Public Campsite	-1.397, 35.004
Narok	63	Mara Conservancy, Iseiya Public Campsite	-1.401, 35.018
Narok	64	Mara Conservancy, Mara River	-1.331, 34.994
Narok	65	Mara Conservancy, Ngiro-Are Anti-Poaching Unit Station	-1.414, 34.794
Narok	66	Masai Mara National Reserve, Keekorok Lodge	-1.590, 35.234
Nyeri	67	Aberdares National Park, Ruhuruini Campground	-0.388, 36.818
Nyeri	68	Mount Kenya National Park, Kisio Munyao Campground	-0.176, 37.147
Nyeri	68	Mount Kenya National Park, Narumoru Gate	-0.175, 37.143
Samburu	69	Samburu National Game Reserve, Larsen’s Camp	0.572, 37.572
Samburu	70	Samburu National Game Reserve, Samburu Game Lodge	0.572, 37.537
Samburu	70	Samburu National Game Reserve, Vervet Campsite	0.567, 37.536
Taita-Taveta	71	Lake Jipe, Mt Kilimanjaro	-3.56, 37.75
Taita-Taveta	72	Marungu Cave	-3.61, 38.74
Taita-Taveta	73	Taita Discovery Centre	-3.706, 38.776
Taita-Taveta	74	Tsavo East National Park, Sala Gate, visitors’ toilets	-3.076, 39.217
Taita-Taveta	75	Tsavo West National Park, Chyulu Gate Ranger Post	-2.902, 38.133
Taita-Taveta	76	Tsavo West National Park, Komboyo Campground	-2.754, 38.115
Taita-Taveta	76	Tsavo West National Park, Park Headquarters	-2.747, 38.131
Taita-Taveta	77	Tsavo West National Park, Shetani Caves	-2.855, 38.001
Tharaka-Nithi	78	Marma Cave	-0.264, 37.687
Trans Nzoia	79	Cherangani Forest Station	1.036, 35.326
Trans Nzoia	80	Mount Elgon National Park, Kitum Cave	1.029, 34.756
Trans Nzoia	80	Mount Elgon National Park, Makingeny Cave	1.036, 34.753
Trans Nzoia	81	Mount Elgon National Park, Rongai Campground	1.031, 34.778
Trans Nzoia	82	Saiwa Swamp National Park, Bushbuck Nature Trail	1.095, 35.118

## ﻿Species accounts

The following species accounts address all known species of nycteribiid bat flies from Kenya, including historical and current records. Each account lists previous Kenyan records (if any) including hosts and distributions, as well as ‘Bats of Kenya’ project records, distribution, hosts, and comments where applicable. We note that Kenyan bat diversity, including phylogeny and taxonomy/nomenclature, is in a fluid state. Ongoing genetic surveys have robustly identified well-supported clades, but some of these clades have not yet been reliably associated with binomial nomenclature. Thus, reference to “clade number” in bat species names follow recent bat publications (e.g., Demos et al. 2018, 2019, 2020, 2023 In Press; Patterson et al. 2020). The previous bat fly records are based on unpublished FMNH records and data compiled by the late T. C. Maa (Bernice P. Bishop Museum, Honolulu, HI USA). Host species identities reported in historical records cannot be verified and are reported as published. We have attempted to update zoological nomenclature as well as names of political units, but older nomenclature that could not be verified or updated is presented in quotation marks.

### ﻿Family Nycteribiidae Samouelle, 1819

**Subfamily Cyclopodiinae Maa, 1965**

**Genus Cyclopodia Kolenati, 1863**

***Cyclopodiagreeffigreeffi* Karsch, 1884**

**Previous records, hosts, distributions.** From *Eidolonhelvum* (Senegal, “French West Africa”, Mali, Liberia, Togolese Republic, Nigeria, Cameroon, Sao Tome, Democratic Republic of the Congo (DRC), Uganda, Sudan, Kenya), *Rousettusaegyptiacus* (DRC, Tanzania), *Epomophorus* sp. (Guinea-Bissau), *Pteropusvoeltzkowi* (Zanzibar). *Macronycterisvittatus* (Ghana), *Nycteristhebaica* (Ghana), *Arvicanthisniloticus* (a rodent!) (Nigeria). From undetermined hosts (Tanzania, DRC, Guinea-Bissau, Sao Tome, Sierra Leone, Nigeria, Dahomey, Ghana, Senegal, Ivory Coast, “French Equatorial Africa”, Bioko).

**Prior Kenyan records.** Kamosi: 6♂, 4♀ from *Eidolonhelvum*, D. E. McInnes, December 1948 (Theodor 1967: 465).

**Bats of Kenya records** (6 records, 26 specimens). Kakamega: 14♂, 12♀ from *Eidolonhelvum* (Mbale), January 2012.

**Distribution.** Generally sub-Saharan West Africa (including Sao Tome), Central Africa, East Africa to Kenya and Tanzania (including Zanzibar).

**Hosts.**Species of the subfamily Cyclopodiinae are largely confined to pteropodid bats. The historical records from *Eidolonhelvum*, and potentially those from species of *Rousettus*, *Pteropus*, and *Epomophorous* are likely legitimate associations. In Kenya, *Cyclopodiagreeffigreeffi* have been collected exclusively from *E.helvum*. Historical records from *Hipposideros*, *Nycteris*, and *Arvicanthis* (grass rat) are likely erroneous associations.

#### ﻿Genus *Dipseliopoda* Theodor, 1955

***Dipseliopodabiannulata* (Oldroyd, 1953)**

**Previous records, hosts, distributions.** From *Myonycterisangolensis* (Cameroon, DRC), *R.aegyptiacus* (DRC, Ghana), *Rousettus* sp. (Kenya), *Epomophorus* sp. (DRC), *Rhinolophuseloquens* or *R.landeri* complex (Sudan), *Tadaridafaini* (DRC). From hosts undetermined (Nigeria, Uganda).

**Kenyan records.** Kakamega: 20 specimens from “*Rousettus* sp.”, Carcasson (Theodor, 1957b: 529).

**Bats of Kenya records** (1 record, 1 specimen). Trans Nzoia: 1♀ from *Epomophoruswahlbergi* (Saiwa Swamp National Park), December 2011.

**Distribution.** Subsaharan West, Central, and East Africa, apparently excluding South African subregion.

**Hosts.** Species of the subfamily Cyclopodiinae are largely limited to pteropodid bats. The historical records from *Rousettusaegyptiacus*, *Myonycterisangolensis*, and *Epomophorous* sp. are presumptively reliable. In Kenya, *Dipseliopodabiannulata* has been collected from *Epomophoruswahlbergi* and *Rousettus* sp. Historical records from species of *Rhinolophus* and *Tadarida* are likely erroneous associations.

***Dipseliopodasetosa* Theodor, 1955**

**Previous records, hosts, distributions.** From *Stenonycterislanosus* (Kenya, Tanzania), *Rousettusaegyptiacus* (Tanzania), *Eidolonhelvum* (Kenya). From host undetermined (Uganda).

**Kenyan records.** Mt. Menengai: 10 specimens from *Stenonycterislanosus*, Hoogstraal (Theodor 1957b: 532).

Ruiru: 2♀ from *Eidolonhelvum*, van Someren (Theodor 1957b: 532).

Ruwenzori: 1♀ from unidentified host, Wollaston (Theodor 1957b: 532).

**Bats of Kenya records** (1 record, 2 specimens). Kakamega: 1♂, 1♀ from *Rousettusaegyptiacus*, Buzambuli Trail 3, Kakamega Forest National Reserve, 31 October 2016.

**Distribution.** Subsaharan Central and East Africa (Uganda, Kenya, Tanzania).

**Hosts.** Species of the subfamily Cyclopodiinae are largely limited to pteropodid bats. The historical records from *Stenonycterislanosus* and *Eidolonhelvum* are likely legitimate.

#### ﻿Genus *Eucampsipoda* Kolenati, 1857

***Eucampsipodaafricana* Theodor, 1955**

**Previous records, hosts, distributions.** From *Rousettusaegyptiacus* (Senegal, Ghana, Sudan, Kenya, Tanzania, South Africa, Sierra Leone, Congo), *Eidolonhelvum* (Cameroon, South Africa). From host undetermined (Malawi). The type series comprised ca 175 specimens from Kenya, Sudan, Congo, Malawi, and South Africa.

**Kenyan records.** Kwale: Shimoni (as *Eucampsipodahyrtli* Kolenati) 4♂, 3♀ from *Rousettusleachi* (=*Rousettusaegyptiacus*), “Grotte A de Shimoni”, Alluaud & Jeannel, 9 November 1911 (Falcoz 1923: 549).

Bahati Cave: 16 specimens (type series) from *Rousettusaegyptiacus*, Garnham (Theodor 1955: 204).

Nakuru: 14 specimens from undetermined host, Zumpt (Theodor 1955: 204).

**Bats of Kenya records** (130 records, 576 specimens). Kilifi: 1♂, 3♀ from *Epomophorouswahlbergi* (Arabuko-Sokoke Forest, Pipit Campsite), October 2012. 3♂ from *Myonycterisangolensis* (Arabuko-Sokoke Forest, Mango orchard 300 m north of Kenya Wildlife Service HQ), May 2006. 107♂, 105♀ from *Rousettusaegyptiacus* (Arabuko-Sokoke Forest, Kenya Wildlife Service HQ; Pipit Campsite; Gedi Ruins; Mango orchard 300 m north of Kenya Wildlife Service HQ; Gede, Watamu Cave; Malindi Marine Park), May 2006 and October 2012.

Kwale: 1♂ from *Miniopterusminor* (Fikirini, Three Sisters, Mdenyenye Cave), September 2012. 42♂, 22♀ from *Rousettusaegyptiacus* (Fikirini, Three Sisters, Mdenyenye Cave), September 2012.

Tharaka-Nithi: 78♂, 91♀ from *Rousettusaegyptiacus* (Marma Cave), December 2012.

Trans Nzoia: 57♂, 66♀ from *Rousettusaegyptiacus* (Mount Elgon National Park, Kitum Cave; Makingeny Cave), December 2011.

**Distribution.** Subsaharan Africa.

**Hosts.** Species of the subfamily Cyclopodiinae are largely limited to pteropodid bats. The historical records from *Rousettusaegyptiacus*, and possibly *Eidolonhelvum* are likely legitimate. The association with *Miniopterusminor* may be accidental, or spillover as this host was roosting in the same cave as *R.aegyptiacus*. In Kenya, associations with *Rousettusaegyptiacus* were by far the most common (mean intensity: 4.47, prevalence: 0.64 based on 568 bat flies from 127 hosts).

### ﻿Subfamily Nycteribiinae Westwood, 1835

**Genus *Basilia* Miranda Ribeiro, 1903**

**Subgenus Basilia**

***Basiliaansifera* Theodor, 1956**

**Previous records, hosts, distributions.** From *Afronycterishelios* (Sudan), *A.nanus* (Nigeria, Ivory Coast, Benin, DRC, Sierra Leone, Liberia), *Pipistrellus* sp. (Ivory Coast, Sudan), *Pseudoromiciarendalli* (Gambia, Sudan), *P.tenuipinnis* (Sierra Leone), *Scotophilus* sp. (Ghana), *Chaerephonpusillus* (mixed with *Pipistrellusnanus* = *Afronycterisnanus*) (Ivory Coast). From hosts undetermined (DRC, Malawi).

**Bats of Kenya records** (5 records, 9 specimens). Meru: 1♂, 1♀ from *Nycticeinopsschlieffeni* (Meru National Park, Kinna), January 2013. Samburu: 2♂, 2♀ from *Nycticeinopsschlieffeni* (Samburu National Game Reserve, Samburu Game Lodge), January 2013. 2♂, 1♀ from *Scotoecushirundo* (Samburu National Game Reserve, Vervet Campsite), January 2013.

**Distribution.** Subsaharan Africa, especially West Africa.

**Hosts and comments.***Basiliaansifera* has been reported from a variety of host bats, including species of *Afronycteris* and *Pseudoromicia*. In Kenya, most specimens were associated with *Nycticeinopsschlieffeni* and *Scotoecushirundo*. This is the first record of *B.ansifera* from Kenya.

***Basiliarobusta* Theodor, 1956**

**Previous records, hosts, distributions.** From *Pipistrelluskuhli* (Zimbabwe), *Afronycterisnanus* (Ethiopia, DRC), *Laephotiscapensis* (Sierra Leone), *Pseudoroemeciatenuipinnis* (DRC), *Eptesicus* sp. (Nigeria), from hosts undetermined (Uganda, Ethiopia, Angola). There are so few records from each reported host species that it is difficult to determine a primary host based on historical records.

**Bats of Kenya records** (35 records, 61 specimens). Kisumu: 3♂, 1♀ from *Pseudoromicianyanza* (Kisumu Impala Sanctuary, State Lodge campsite), January 2012.

Laikipia: 7♂, 5♀ from *Laephotiscapensis* (Lolldaiga Hills Ranch, Farm house; Simba Campsite; Munanda Dam; Ol Jogi Wildlife Conservancy, Kiboko Campsite), July and August 2014. 6♂, 8♀ from undetermined *Neoromicia* sp. (Lolldaiga Hills Ranch, Munanda Dam; Valley Dam), July 2014. 2♂, 1♀ from undetermined *Pipistrellus* sp. (*aero* or *hesperidus*) (Ol Jogi Conservancy, Kiboko Campsite), August 2014. 6♂, 10♀ from Pipistrelluscf.hesperidus (Lolldaiga Hills Ranch, Kambi Dam; Main house; Munanda Dam; Shaita Dam; Valley Dam), July 2014. 1♂ from *Scotophilus* clade 2 (Lolldaiga Hills Ranch, Shaita Dam), July 2014.

Marsabit: 2♂, 4♀ from undetermined *Pipistrellus* sp. (*aero* or *hesperidus*) (Marsabit National Park and Reserve, 12.09 km SW of campground; 6.07 km SW of campground), July 2015. 2♂, 1♀ from Pipistrelluscf.hesperidus (6.07 km SW of campground), July 2015.

Narok: 1♂ from Pipistrelluscf.hesperidus (Masai Mara National Reserve, Mara Simba Lodge), January 2014.

Samburu: 1♂ from *Laephotiscapensis* (Samburu National Game Reserve, Vervet Campsite), January 2013.

**Distribution.** Subsaharan Africa, apparently excluding South Africa.

**Hosts and comments.***Basiliarobusta* has previously been reported from a variety of host bats, including species of *Pipistrellus* and *Eptesicus*. In Kenya, 61 specimens were collected, largely associated with bat species in the genera *Laephotis*, *Neoromicia*, *Pseudoromicia*, and *Pipistrellus*. The single fly specimen from *Scotophilus* may be an accidental association. This is the first record of *B.robusta* from Kenya.

### ﻿Subgenus Tripselia Scott, 1917

***Basiliablainvilliiblainvillii* (Leach, 1817)**

**Previous records, hosts, distributions.** From *Taphozousmauritianus* (Sierre Leone, Ivory Coast, Cameroon, DRC, Angola, Sudan, Tanzania, Mozambique, Assumption Islands), *Taphozousperforatus* (Egypt), *T.peli* (=*Saccolaimuspeli*; DRC), *Nycteristhebaica* (Tanzania), *Pteropus* sp. (Comoros), *Rousettus* sp. (Benin, Kenya), *Epomophoruslabiatus* (Tanzania), from hosts undetermined (Gold Coast).

**Kenyan records.** Kiambu: “Kyambu,” 3♀ from undetermined host, Garnham (Theodor 1956: 359).

**Distribution.** Subsaharan Africa and Egypt.

**Hosts.***Basiliablainvilliiblainvillii* has previously been reported from a variety of host bats, including species of *Taphozous*, *Nycteris*, and several genera of pteropid bats. Theodor (1956) stated that this species was largely associated with *Taphozousmauritianus*.

### ﻿Subgenus Paracyclopodia Scott, 1917

***Basiliabouvieri* (Falcoz, 1924)**

**Previous records, hosts, distributions.** From *Scotophilusleucogaster* (Senegal, Uganda), “*S.nigrita*” or “*S.dinganii*” (Senegal, Sierra Leone, Ghana, Sudan), *Scotophilus* sp. (Sudan), *Eptesicusphasma* (= *Pseudoromiciarendalli*) (Sudan). From hosts undetermined (Tanzania).

**Bats of Kenya records** (7 records, 25 specimens). Narok: 1♂ from *Scotophilus* clade 4, January 2014.

Marsabit: 5♂, 19♀ from *Scotophilusandrewreborii* (Marsabit National Park and Reserve, 12.09 km SW of campground; campground near headquarters), July 2015.

**Distribution.** Subsaharan Africa, apparently excluding South Africa.

**Hosts and comments.***Basiliabouvieri* has previously been reported from a variety of host bats, primarily species of *Scotophilus*. In Kenya, 25 specimens were collected and all but one was associated with *S.andrewreborii*. This is the first record of *B.bouvieri* from Kenya.

#### ﻿Genus *Nycteribia* Latreille, 1796

**Subgenus Nycteribia**

***Nycteribialatitergum* Theodor, 1957**

**Previous records, hosts, distributions.** Mt. Menangai near Nakuru: ca 30 specimens (type series) from mixed samples of *Miniopterusarenarius* and *Myotistricolor*, Hoogstraal, 8 June 1948 (Theodor 1957b: 471).

**Kenyan records.** Previously known only from the type series (see above).

**Bats of Kenya records** (5 records, 7 specimens). Laikipia: 2♀ from *Laephotiscapensis* (Lolldaiga Hills Ranch, Munanda Dam; Gilgil, Diatomite Cave), July 2014.

Nakuru: 3♂, 2♀ from *Myotistricolor* (Menengai Crater, Mau Mau Cave), June 2014.

**Distribution.** Kenya (Theodor 1967 listed “East Africa”).

**Hosts.***Nycteribialatitergum* has been previously reported from *Miniopterusarenarius*. Specimens collected during the Bats of Kenya project were found in association with *Myotistricolor* and *Laephotiscapensis*.

***Nycteribiaschmidliiscotti* Falcoz, 1923**

**Previous records, hosts, distributions.** From *Miniopterusinflatus* (Cameroon, French Guinea, DRC), *M.minor* (Kenya, DRC), “*M.schreibersii*” (Sudan, Kenya, Mozambique, South Africa), *Miniopterus* sp. (Sudan), *Afronycterisnanus* (Cameroon, Nigeria, South Africa), *Eptesicus* sp. (Sudan), *Rhinolophusclivosusaugur* (South Africa), *R.hildebrandtii* (DRC), from mixture of *R.capensis* and *Laephotiscapensis* (South Africa), *Hipposideroscaffer* (DRC), *Triaenopsafer* (Mozambique), *Mopsniveiventer* (DRC), *Nycteriscapensis* (=*N.thebaica*; Zimbabwe), from undetermined hosts (Zambia, Sao Tome Island).

**Kenyan records.** Kwale: Shimoni (as *Nycteribiascotti* Falcoz) 3♂, 2♀ from *Miniopterusminor*; 1♂, 1♀ from *Hipposideroscaffer*, “Grotte A de Shimoni”, Alluaud & Jeannel, 9 November 1911 (Falcoz 1923: 548).

Ngong near Mt. Elgon: 14 specimens from *Miniopterus* sp., Cade (Theodor 1957b: 465).

Mt. Elgon: 60 specimens from “*M.schreibersii*” [likely *Miniopterusinflatus* or *M.africanus*], Edwards (Theodor 1957b: 465).

Mt. Menangai: 6 specimens from unknown host, Hoogstraal (Theodor 1957b: 465).

Kapretwa, Kitale: 6 specimens from “*M.schreibersii*”, Hopkins (Theodor 1957b: 466).

**Bats of Kenya records** (177 records, 408 specimens). Kajiado: 35♂, 36♀ (Mount Suswa, Cave 14C; Cave 18A) from *Miniopterusafricanus*, August 2011.

Kakamega: 40♂, 55♀ from *Miniopterusinflatus* (Kakamega Forest, Lirhanda Hill Cave; Mahiakalo Cave), January 2012.

Kilifi: 9♂, 9♀ from *Coleuraafra* (Watamu, Makuruhu Cave), October 2012. 2♂ from *Miniopterus* clade 2 or 5 (Watamu, Makuruhu Cave), October 2012.

Kwale: 1♂, 1♀ from *Coleuraafra* (Mwaluganje Community Elephant Sanctuary, Ngomeni Cave), September 2012. 74♂, 63♀ from *Miniopterusminor* (Fikirini, Pare Cave; Three Sisters, Kisimani Cave; Three Sisters, Mdenyenye Cave; Three Sisters, Pangani Cave; Mwaluganke Community Elephant Sanctuary, Ngomeni Cave), September 2012. 2♂ from Miniopteruscf.villiersi (Mwaluganje Community Elephant Sanctuary, Ngomeni Cave), September 2012.

Laikipia: 9♂, 12♀ from *Miniopterus* clade 7 (Lolldaiga Hills Ranch, Simba Campsite Dam), July 2014.

Nakuru: 6♂, 3♀ from *Miniopterus* clade 1 (Gilgil, Kariandusi Mines; Menengai Crater, Mau Mau Cave), January, June, and August 2014. 11♂, 7♀ from *Miniopterus* clade 1 or 4 (Gilgil, Kariandusi Mines; Menengai Crater, Mau Mau Cave), June and August 2014. 1♀ from *Miniopterus* clade 4 (Menengai Crater, Mau Mau Cave), August 2014. 1♀ from Rhinolophuscf.landeri (Gilgil, Kariandusi Mines), June 2014.

Nyeri: 2♀ from *Miniopterus* clade 1 (Mount Kenya National Park, Narumoru Gate), January 2013.

Taita-Taveta: 4♂, 7♀ from *Miniopterus* clade 5 (Mount Kilimanjaro, Lake Jipe), October 2012. 3♂, 3♀ from *Miniopterus* clade 7 (Marungu Cave), April 2006.

Trans Nzoia: 5♂, 8♀ from *Miniopterus* clade 1 (Mount Elgon National Park, Kitum Cave; Makingeny Cave), December 2011.

**Distribution.** Subsaharan Africa.

**Hosts.***Nycteribiaschmidliiscotti* has been previously reported from a variety of bats, including species of both Yinpterochiroptera (*Rhinolophus*, *Hipposideros*, and *Triaenops*), and two of three superfamilies of Yangochiroptera (*Miniopterus*, *Pipistrellus*, *Eptesicus*, *Mops* and *Nycteris*). However, the recent collection efforts in Kenya recovered 408 specimens, the vast majority of which were associated with various species/clades of *Miniopterus* and to a far lesser extent *Coleuraafra*. The single specimen collected from Rhinolophuscf.landeri may well represent an erroneous record.

#### ﻿Genus *Penicillidia* Kolenati, 1863

**Subgenus Penicillidia**

***Penicillidiafulvida* (Bigot, 1885)**

**Previous records, hosts, distributions.** From “*Miniopterusschreibersii*” (South Africa, Mozambique, Kenya), *M.inflatus* (DRC, Cameroon), *Miniopterus* sp. (Sudan), *Myotistricolor* (South Africa, Kenya), *Rhinolophusblasii* (Yemen), *R.clivosus* (South Africa), *R.eloquens* (Sudan), *R.foxi* (=*R.fumigatus*; Cameroon), *R.hildebrandtii* (Tanzania), from *R.keniensis* (=*R.clivosus*; Kenya), *Rhinolophus* sp. (South Africa, DRC, Sudan, Benin, “*Hipposideroscaffer*” (Mozambique, Kenya, DRC), *Nycteristhebaica* (South Africa, Mozambique, DRC), *Coleuragallarum* (Sudan), *Eidolonhelvum* (South Africa).

**Kenyan records.** Kericho: 1♀ from “*Hipposideroscaffer*”, Dobbs (Theodor 1957b: 513).

Mt. Elgon: 21 specimens from “*M.schreibersii*”, 1♀ from *Rhinolophusclivosus*, Edwards (Theodor 1957b: 513).

Mt. Menengai, Rift Valley: 10 specimens from “*M.schreibersii*” and *Myotistricolor*, Hoogstraal (Theodor 1957b: 513).

**Bats of Kenya records** (58 records, 65 specimens). Kakamega: 2♂, 4♀ from *Miniopterusinflatus* (Kakamega Forest, Lirhanda Hill Cave; Mahiakalo Cave), January 2012 and September 2014.

Kwale: 1♂, 5♀ from *Miniopterusminor* (Fikirini, Pare Cave; Three Sisters, Kisimani Cave; Three Sisters, Mdenyenye Cave), September 2012. 1♀ from Miniopteruscf.villiersi (Mwaluganje Community Elephant Sanctuary, Ngomeni Cave), September 2012. 1♂ from *Nycteristhebaica* clade 4 (Shimba Hills National Reserve, Sable Bandas), October 2012. 1♀ from *Rhinolophusfumigatus* clade 8 (Fikirini, Pare Cave), September 2012. 1♀ from *Taphozoushildegardeae* (Mwaluganje Community Elephant Sanctuary, Ngomeni Cave), September 2012. 1♀ from *Triaenopsafer* (Fikirini, Three Sisters, Mdenyenye Cave), September 2012.

Marsabit: 1♀ from Rhinolophuscf.landeri (Marsabit National Park and Reserve, campground near headquarters), July 2015. 1♂ from *Rhinolophusfumigatus* clade 2 or 3 (Marsabit National Park and Reserve, 6.07 km SW campground near headquarters), July 2015. 5♀ from *Rhinolophusfumigatus* clade 3 (Marsabit National Park and Reserve, campground near headquarters; 6.07 km SW of campground; 1.3 km SE of campground), July 2015.

Nakuru: 4♂, 3♀ from *Miniopterus* clade 1 (Gilgil, Kariandusi Mines), January and August 2014. 2♂, 9♀ from *Miniopterus* clade 1 or 4 (Gilgil, Kariandusi Mines; Menengai Crater, Mau Mau Cave), June and August 2014. 1♂, 1♀ from *Miniopterus* clade 4 or 7 (Gilgil, Pipeline Cave), August 2014. 1♀ from *Miniopterusafricanus* (Gilgil, Kariandusi Mines), September 2014. 3♂, 7♀ from *Myotistricolor*, June and August 2014 (Menengai Crater, Mau Mau Cave; Soysambu Conservancy, Monkey Bridge Campsite). 1♂ from Rhinolophuscf.landeri (Gilgil, Kariandusi Mines), August 2014. 2♂ from *Rhinolophusclivosus* clade 2 (Gilgil, Kariandusi Mines), January and September 2014. 1♀ from *Rhinolophusfumigatus* clade 4 (Gilgil, Pipeline Cave), September 2014.

Taita-Taveta: 1♂, 4♀ from *Coleuraafra* (Marungu Cave; Tsavo West National Park, Shetani Caves), April and May 2006. 1♂ from *Miniopterus* sp. (Marungu Cave), April 2006.

**Distribution.** Subsaharan Africa, Arabian Peninsula (Yemen).

**Hosts and comments.***Penicillidiafulvida* has been reported in association with a remarkable variety of bats in the families Pteropodidae, Rhinolophidae, and Hipposideridae (Suborder Yinpterochiroptera) as well as Vespertilionidae, Emballonuridae, and Nycteridae (Suborder Yangochiroptera). Theodor (1967) remarked that this species is apparently quite unspecific [to host species of bats] and had been reported from 14 species, 7 genera, and 5 families of bat. The 65 specimens of *P.fulvida* collected recently in Kenya were also recovered from a wide variety of host bats, with little evidence of population structure among the specimens (Verrett et al. 2022). *Penicillidiafulvida* is a rarity among bat flies in its demonstrable lack of host specificity, even with respect to families and suborders.

***Penicillidiapachymela* Speiser, 1901**

**Previous records, hosts, distributions.** From “*Hipposideroscaffer*” (Mozambique, DRC, Tanzania, Zambia), *Hipposideros* sp. (DRC, Cameroon), *Rhinolophushildebrandtii* (Mozambique), *R.landeri* (DRC, Cameroon), from mixture of *R.eloquens* and *R.lobatus* (Sudan), *Nycteristhebaica* (Mozambique), *Nycteris* sp. (Tanzania), from undetermined hosts (Somalia, “French Equatorial Africa”).

**Kenyan records.** Nairobi: 1♂ from undetermined host, February 1912 (Theodor 1967: 374).

Ngong hills (near Nairobi): 1♂ from undetermined host, 19 September 1934 (Theodor 1967: 374).

Tana Bridge: 2♂ from undetermined hosts, 1 February 1948 (Theodor 1967: 374).

**Bats of Kenya records** (1 record, 1 specimen). Nakuru: 1♂ from *Hipposideroscaffer* clade 1 (Lake Nakuru National Park, Lion Hill Cave), August 2011.

**Distribution.** Subsaharan Africa, apparently excluding South Africa.

**Hosts.** This rarely encountered species has been reported in association with a variety of species. The single specimen collected during the ‘Bats of Kenya’ survey was associated with *Hipposideroscaffer* clade 1. Too few specimens exist to determine whether *P.pachymela* exhibits the broad host range seen in *P.fulvida*.

#### ﻿Genus *Phthiridium* Hermann, 1804

***Phthiridiumhoogstraali* (Theodor, 1957)**

**Previous records, hosts, distributions.** From *Rhinolophuseloquens* (Sudan), *R.hildebrandtii* (DRC), *Rhinolophus* sp. (Sudan).

**Bats of Kenya records** (45 records, 130 specimens). Kisumu: 1♂, 2♀ from *Rhinolophusfumigatus* clade 1 (Kisumu Impala Sanctuary, State Lodge Campsite), January 2012.

Laikipia: 1♂, 3♀ from *Rhinolophusfumigatus* clade 1 (Lolldaiga Hills Ranch, Simba Campsite Dam), July 2014.

Nakuru: 8♂, 7♀ from *Rhinolophusfumigatus* clade 1 (Lake Nakuru National Park, Lion Hill Cave), August 2011 and January 2012. 24♂, 59♀ from *Rhinolophusfumigatus* clade 1 or 4 (Lake Nakuru National Park, Lion Hill Cave), August 2011 and January 2012. 12♂, 13♀ from *Rhinolophusfumigatus* clade 4 (Gilgil, Pipeline Cave; Lake Nakuru National Park, Lion Hill Cave), August 2011, January 2012, and August 2014.

**Distribution.** Subsaharan Africa, excluding South Africa.

**Hosts and comments.***Phthiridiumhoogstraali* has previously been reported from at least two species of *Rhinolophus*. In Kenya, 130 specimens were collected and all belonged to clades identified as *Rhinolophusfumigatus*. This is the first record of *Phthiridiumhoogstraali* from Kenya.

***Phthiridiuminopinata* (Theodor, 1957)**

**Previous records, hosts, distributions.** From *Rhinolophusalcyone* (Cameroon)

**Bats of Kenya records** (1 record, 2 specimens). Kakamega: 1♂, 1♀ from *Hipposiderosbeatus* clade 1 (Kakamega Forest, Ikhondo campground), January 2012.

**Distribution.** Subsaharan Africa (Cameroon, Kenya).

**Hosts and comments.***Phthiridiuminopinata* is apparently scarce in nature and has previously been reported from *Rhinolophusalcyone*, which is distributed in west and central Africa. In Kenya, two specimens were collected from *Hipposiderosbeatus* in Kakamega Forest (western Kenya), the easternmost extension of Africa’s equatorial rainforests. This is the first record of *Phthiridiuminopinata* from Kenya.

***Phthiridiumrhodesiense* (Theodor, 1957)**

**Previous records, hosts, distributions.** From *Rhinolophushildebrandtii* (Zimbabwe), *R.darlingi* (Zimbabwe), *Nycteristhebaicacapensis* (Zimbabwe), from undetermined host (Malawi).

**Bats of Kenya records** (3 records, 4 specimens). Makueni: 2♂ from *Rhinolophushildebrandtii* clade 1 (Chyulu Hills National Park, Kisula Cave), May 2006.

Taita-Taveta: 2♀ from *Rhinolophushildebrandtii* clade 1 (Tsavo West National Park, Shetani Caves), May 2006.

**Distribution.** Subsaharan east Africa (Kenya, Malawi, Zimbabwe).

**Hosts and comments.** The historical records of *Phthiridiumrhodesiense* have largely been associated with species of *Rhinolophus*. In Kenya, four specimens were collected from two individuals of *Rhinolophushildebrandtii*. This is the first record of *Phthiridiumrhodesiense* from Kenya.

***Phthiridiumscissum* (Speiser, 1901)**

**Previous records, hosts, distributions.** From *Rhinolophuscapensis* (South Africa), *R.darlingi* (South Africa), *R.hildebrandtii* (Mozambique), *R.clivosus* (Namibia, South Africa), from mixture of *R.eloquens*, *Hipposideroscaffer*, and *Nycteriscapensis* (Namibia).

**Bats of Kenya records** (56 records, 134 specimens). Marsabit: 3♂, 4♀ from *Rhinolophusfumigatus* clade 2 (Marsabit National Park and Reserve, 12.09 km SW of campground near headquarters; 6.07 SW of campground near headquarters), July 2015. 36♂, 44♀ from *Rhinolophusfumigatus* clade 2 or 3 (Marsabit National Park and Reserve, 1.3 km SE of campground and headquarters; 12.09 km SW of campground near headquarters; 6.07 km SW of campground near headquarters), July 2015. 19♂, 20♀ from *Rhinolophusfumigatus* clade 3 (Marsabit National Park and Reserve, 1.3 km SE of campground near headquarters; 12.09 km SW of campground near headquarters; 6.07 km SW of campground near headquarters; campground near headquarters), July 2015.

Nakuru: 2♂, 4♀ from *Rhinolophusclivosus* clade 2 (Gilgil, Kariandusi Mines), January 2014.

Taita-Taveta: 2♀ from *Rhinolophusfumigatus* clade 2 (Tsavo West National Park, Shetani Caves), May 2006.

**Distribution.** Subsaharan Africa (Kenya, South Africa, Namibia, Mozambique).

**Hosts and comments.** The historical records of *Phthiridiumscissum* have largely been associated with species of *Rhinolophus*, but there are records from species of *Hipposideros* and *Nycteris*. In Kenya, 134 specimens were collected, all from clades referred to *Rhinolophusfumigatus*. These are the first records of *Phthiridiumscissum* from Kenya.

***Phthiridiumtectum* (Theodor, 1957)**

**Previous records, hosts, distributions.** From *Miniopterusarenarius* (reported as *Rhinolophusschreibersiiarenarius* (Kenya), *Miniopterus* sp. (Kenya), *Eptesicus* sp. (Sudan), *Rhinolophusdarlingi* (South Africa), *Rhinolophusdeckeni* (Uganda), *Rhinolophus* sp. (Tanzania), *Hipposideroscaffer* (Zimbabwe).

**Kenyan records.** Ngong near Mt. Elgon: 1♀ (holotype) from *Miniopterus* sp. (Theodor 1957b: 485).

Kapretwa, Kitale: 1♀ from *Miniopterusarenarius*, 15 January 1957 (Theodor 1967: 178).

**Distribution.** Subsaharan Africa (Kenya, Sudan, Uganda, Tanzania, Zimbabwe, South Africa).

**Hosts and comments.** The historical records of *Phthiridiumtectum* hav been associated with a variety of bats in the genera *Miniopterus*, *Rhinolophus*, *Eptesicus*, and *Hipposideros*. This species is apparently rare in Kenya, and no recent collections were made during the ‘Bats of Kenya’ survey.

***Phthiridium* sp. nov. from *Rhinolophusclivosus***

**Notes.** Three male specimens collected from two “Rhinolophusclivosus 2” represent a putative undescribed species. Given the lack of female specimens and that many diagnostic characteristics are associated with the female abdomen, we decline to describe this new species based on the inadequate material presently available.

**Kenyan records.**

**Bats of Kenya records** (2 records, 3 specimens). Nakuru: 3♂ from *Rhinolophusclivosus* clade 2 (Gilgil, Kariandusi Mines), June 2014.

**Distribution.** Known only from Kenya.

**Hosts.** The three known specimens were all collected from a *Rhinolophusclivosus* clade 2 individual, at the Kariandusi mines near Gilgil.

## ﻿Discussion

This effort represents the most extensive catalog to date of nycteribiid biodiversity in Kenya, and one of the most thorough summaries of nycteribiid diversity in the Afrotropical region. The ‘Bats of Kenya’ survey documented 7 nycteribiid species previously unknown from Kenya (*Basiliaansifera*, *B.bouvieri*, *B.robusta*, *Phthiridiumhoogstraali*, *P.inopinata*, *P.rhodesiense*, and *P.scissum*), as well as three males of a putative new species in the genus *Phthiridium*. These records raise the richness of nycteribiid bat flies cataloged from Kenya to 17 species in 7 genera (Appendix 1).

The geographic sampling distribution of bats across Kenya was reasonably thorough with respect to biodiversity centers. Localities sampled in the ‘Bats of Kenya’ survey were concentrated in the tropical forests and woodlands containing much of Kenya’s bat biodiversity; gaps in coverage comprise much drier parts and brushlands in northern Kenya which are depauperate in bats. Notable exceptions are some stretches of coastal forest at or near the Somali border, including the Boni and Dodori National Reserves, which were not sampled due to security concerns. Kenya’s coastal forests are recognized as global biodiversity hotspots with high degrees of endemism (Myers et al. 2000), and further sampling efforts should target this area when it is safe to do so. Further, although the unsampled northwestern and northeastern regions of Kenya are composed mainly of savanna or arid habitat with relatively few bat species (Herkt et al. 2016), elevated areas can intercept orographic precipitation and may support woodlands containing unique bat and bat fly communities (Monadjem and Reside 2008). They surely warrant future survey attention.

The most biodiverse habitats in Kenya are also those most prone to habitat loss, modification and fragmentation, as areas with higher water availability are attractive for anthropogenic use in an overall arid country (Bennun and Njoroge 2000). It is crucial for Kenyan biodiversity to be more thoroughly explored as natural areas globally are depleted by habitat loss. Moreover, habitat fragmentation can affect the size and isolation of populations (e.g., Webala et al. 2019), influencing transmission dynamics of vector-borne diseases in patterns mediated by host and parasite ecology (Suzán et al. 2012). Land conversion and habitat fragmentation, particularly in highly biodiverse areas, also increases the probability of human-wildlife interaction and can facilitate the spread of zoonotic diseases (Johnson et al. 2020). Nycteribiid bat flies are vectors of bacterial pathogens in genus *Bartonella* (Wilkinson et al. 2016) and of haemosporidian parasites of bats (Megali et al. 2010; Lutz et al. 2016). Bat flies are also becoming increasingly linked to viral pathogens related to bat-associated zoonoses, though their role as potential vectors or principal carriers of such diseases remains unclear (Bennett et al. 2020, Ramírez-Martínez et al. 2021). As the role of bat flies in disease transmission is further elucidated, bat fly diversity must be understood at a fundamental level in areas where it remains largely unexplored. The need to investigate bat fly vector potential and diversity is especially salient in continental Africa, which harbors 22.4% of all bat biodiversity (MDD 2022) and accounted for more than half of all emerging infectious disease outbreaks between 1996 and 2009 (Chan et al. 2010).

## ﻿Additional information

### Author ORCIDs

Carl W. Dick https://orcid.org/0000-0002-7491-6084

Paul W. Webala https://orcid.org/0000-0003-0576-8935

Bruce D. Patterson https://orcid.org/0000-0002-2249-7260

### Data availability

All of the data that support the findings of this study are available in the main text or Supplementary Information.

## ﻿Appendix 1

List of 17 nycteribiid bat fly species known from Kenya. New country records from ‘Bats of Kenya’ surveys are denoted by an asterisk.

*Cyclopodiagreeffigreeffi* Karsch, 1884

*Dipseliopodabiannulata* (Oldroyd, 1953)

*Dipseliopodasetosa* Theodor, 1955

*Eucampsipodaafricana* Theodor, 1955

**Basiliaansifera* Theodor, 1956

**Basiliarobusta* Theodor, 1956

*Basiliablainvilliiblainvillii* (Leach, 1817)

**Basiliabouveri* (Falcoz, 1924)

*Nycteribialatitergum* Theodor, 1957

*Nycteribiaschmidliiscottii* Falcoz, 1923

*Penicillidiafulvida* (Bigot 1885)

*Pencillidiapachymela* Speiser, 1901

* *Phthiridiumhoogstraali* (Theodor, 1957)

* *Phthiridiuminopinata* (Theodor, 1957)

* *Phthiridiumrhodesiense* (Theodor, 1957)

* *Phthiridiumscissum* (Speiser, 1901)

*Phthiridiumtectum* (Theodor, 1957)

* *Phthiridium* sp. nov. ex. *Rhinolophusclivosus*
